# The effects of compulsory health insurance on birth outcomes: evidence from China’s UEBMI scheme

**DOI:** 10.1186/s12913-019-4657-1

**Published:** 2019-11-01

**Authors:** Di Tang, Xiangdong Gao, Peter C. Coyte

**Affiliations:** 1 Shanghai First Maternity and Infant Hospital, Tongji University School of Medicine, No. 2669 Gaoke West Road, Pudong New Area, Shanghai, 201204 China; 2 School of Public Administration, East China Normal University, Zhongshan Rd, Shanghai, 200062 China; 3 Institute of Health Policy, Management, and Evaluation, University of Toronto, 155 College Street, Suite 425, Toronto, ON M5T 3M6 Canada

**Keywords:** Health insurance, Birth outcomes, Propensity score matching, Infant health

## Abstract

**Background:**

Despite extensive research concerning the impact of health insurance on the advancement of infant health in developed countries, few studies have adjusted their results for potential confounding due to adverse selection in insurance coverage, wherein those who anticipate a need for health services tend to be the ones that acquire insurance. The presence of compulsory health insurance in China, such as the Urban Employee Basic Medical Insurance (UEBMI) scheme may provide an opportunity to estimate the effect of health insurance on infant health, by reducing the endogeneity problem into insurance due to the adverse selection. The objective is to assess the relationship between UEBMI and infant health outcomes in one sizeable municipal-level obstetrics hospital in Shanghai, East China.

**Methods:**

Medical records data from the Shanghai First Maternity and Infant Hospital from January 1, 2013 to April 30, 2019 were used to form an analysis dataset of 160,429 live births which was comprised of Shanghai residents with UEBMI coverage (*n* = 101,153) and women without any insurance coverage (*n* = 59,276). A propensity score matching approach using conjoint quantile regression and probit regression models was used to eliminate latent endogeneity of UEBMI coverage in order to garner robust results. Further analysis stratified by maternal migrant status was conducted to further assess the sensitivity of the findings to distinct patient subgroups.

**Results:**

The UEBMI scheme was shown to be associated with improvements in infant birth outcomes. The scheme was associated with: an increase in birth weight of about 30 g (*p* <  0.001, 95% CI 23.908–35.295). This finding was evident in other five different birth outcomes (premature birth, low birth weight, very low birth weight, low Apgar score, and an abnormal health condition at birth). After stratifying by migrant status, the UEBMI was shown to have a greater effect on migrants compared to local residents of Shanghai.

**Conclusions:**

Our findings suggest that health insurance coverage for pregnant women, especially for migrants, has the potential to significantly and directly improve infant health outcomes. Further research is required to determine whether these findings can be replicated for other Chinese jurisdictions.

## Background

Health insurance has the potential to improve access to health care services by removing financial barriers to utilization. In turn, such insurance has the potential to improve infant health. Despite extensive research concerning the impact of health insurance on the advancement of infant health [1–4], there exists a paucity of research for low-to-middle-income countries, such as China. Moreover, while there are studies of the association between various dimensions of health insurance and infant health outcomes, few of these studies have addressed the potential confounding of this relationship as the decision to acquire health insurance is a choice that may be associated with an underlying need for health care. Consequently, the observed association between the occurrence of insurance and positive health outcomes may underestimate the contribution of insurance. The presence of compulsory health insurance in China, such as the Urban Employee Basic Medical Insurance (UEBMI) scheme, which was introduced in 1998, may provide an opportunity to estimate the effect of health insurance on infant health by reducing the potential confounding due to adverse selection in insurance coverage.

Reductions in financial barriers to accessing health care services have been argued as one of the most important mechanisms by which population health may be advanced, in general, and infant health improved, in particular [5]. While one of the critical mechanisms by which financial barriers may be reduced is through the provision of health insurance [1], there is conflicting evidence of the association between insurance coverage and improvements in infant health [1–3, 6]. One potential explanation for this ambiguity in the empirical literature is one of confounding. Specifically, a common occurrence in insurance markets is the potential for adverse selection wherein those that purchase insurance tends to be individuals with the greatest need for health care. If individuals with lower health status were more likely to have insurance than those without such insurance, it is quite possible to observe a negative relationship between insurance coverage (as a marker for poor health status) and health outcomes. Consequently, to capture the possible unbiased effect of insurance coverage on health outcomes, it is important to control for potential confounding. Without addressing this issue, the empirical estimates of the relationship between insurance coverage and infant health outcomes are likely to be biased.

The introduction of the Urban Employee Basic Medical Insurance (UEBMI) scheme in 1998 for employed urban residents in China may provide an opportunity to assess the effect by reducing the potential confounding due to adverse selection in insurance coverage on health outcomes as the scheme was compulsory. Coverage was financed through a payroll tax of 6% on employers and 2% on employees with the balanced paid by the government [7, 8]. A claimant for unemployment insurance could still receive UEBMI coverage for up to 24 months if the person was temporarily out-of-work.

In China, the health of migrants tends to be worse than that of urban residents, especially for maternal and infant health, since migrants often are excluded from urban services [9]. Migrants could have UEBMI coverage if they obtain a job of sufficient standing in cities, which in turn may improve their health. Of course, not all migrants have this opportunity, and consequently, health outcomes would vary across migrants.

This study aims to estimate the effect of the UEBMI scheme on infant health outcomes in China. To achieve our research objective, we used a propensity score matching method to eliminate potential endogeneity bias. Six separate birth outcomes were assessed in our evaluation: birth weight; and the incidence of having a premature birth, a child with low birth weight, a child with very low birth weight, a low Apgar score, and the incidence of having a child with an abnormal health condition at birth.

In the next section, we offer a brief overview of the literature concerning the relationship between insurance and infant health outcomes. Data and methods are described in Section 3. Section 4 summarizes our results and considers the robustness and sensitivity of our findings. Our findings are discussed in Section 5 in the context of the literature. Section 6 offers a brief set of conclusions and policy implications.

## Literature review

There are various ways in which health insurance has been shown to impact infant health. First, health insurance tends to increase both the quantity [5] and the quality of health service use [10]. These responses to health insurance may improve infant health [5]. Second, health care use (and in turn, exposure to the advice of health professionals) may result in improvements in healthy behaviors, such as dietary change, that may assist in their control over anemia, gestational diabetes and pregnancy-induced hypertension [11]. Of course, health insurance coverage may have the opposite effect; by protecting individuals from the financial consequences of poor health behaviors, health insurance coverage provides incentives to engage in behaviors that may increase exposure to health care use, the so-called “moral hazard effect” [12]. This effect may result in less preventative health practices if individuals know that the financial consequences of the resulting poor health were protected by insurance coverage. Third, health insurance coverage tends to reduce the financial burden of health service utilization, and accordingly, lowers financial stress and may enhance disposable household income [10]. These combined effects will tend to improve health during pregnancy and result in positive health outcomes thereafter.

While the theory underlying the effect of health insurance on infant health is well developed, the empirical literature is inconsistent. Using regression analysis, Currie and Gruber (1996) showed that an expansion in Medicaid was associated with a decline in child mortality and a smaller decline in the incidence of low birth weight (LBW) [2]. Hanratty (1996) found that public health insurance in Canada resulted in improvements in infant mortality and a smaller improvement in the incidence of low birth weight (LBW) [6]. In contrast, Howell (2001) conducted a systematic review and found only weak evidence that the expansion of Medicaid improved infant birth outcomes [3]. These inconsistencies in the literature may be due to potential confounding [5]. In terms of East Asia, only four studies have examined the effect of insurance on infant health outcomes. Gruber et al. (2014) examined the 30-Baht program in Thailand and found that it was associated with a reduction in infant mortality [13]. Chou, Grossman, & Liu (2014) conducted a difference in differences (DID) approach and found that the introduction of National Health Insurance in Taiwan led to a drop in post-neonatal mortality for farm households, but not for private-sector households [14]. Chen and Jin (2012) examined the effects of the New Cooperative Medical Scheme (NCMS) in rural China and did not find evidence that this scheme had an impact on child mortality [15]. As mentioned previously, unlike the compulsory Urban Employee Basic Medical Insurance (UEBMI) scheme, the New Cooperative Medical Scheme was voluntary and may have led to biased estimates due to issues of confounding. Tang, Gao, & Fang (2019) used propensity score matching (PSM) methods and found that all types of health insurance coverage in China improved newborn health [16]. However, this study did not control for potential adverse selection into insurance.

With increasingly rich data and improvements in econometric methods, there is the potential to assess the impact of insurance coverage on an array of health outcomes, including infant mortality, birth weight, low Apgar scores, premature birth, gestational weeks, and other birthweight-related outcomes, such as the occurrence of an abnormal health condition after birth as diagnosed by a pediatrician [17]. Among the infant outcomes assessed in the literature, birth weight has been used extensively [18].

There have also been improvements in econometric methods to deal with confounding and other statistical challenges include propensity score matching (PSM), instrumental variables (IV), and the difference in differences (DID) approach. When combined innovative econometric techniques and detailed clinical data have the potential to identify the unique impact of health insurance on infant health outcomes, thereby potentially yielding a more consistent set of results. Using fixed effects (FE) and instrumental variables (IV) methods, Bitler & Currie (2005) demonstrate that participation in the Special Supplemental Nutrition Program for Women, Infants, and Children (WIC) in the US reduced the probability of low birth weight (LBW) and very low birth weight (VLBW) [19]. Joyce, Racine, & Yunzal-Butler (2008) used the difference in differences (DID) approach and reported modest favorable effects of WIC participation on fetal growth [20]. Figlio, Hamersma, & Roth (2009) also used IV methods and found that WIC participation had no effect on mean birth weight (BW) and gestational age (GA) but reduced the likelihood of low birth weight (LBW) and premature birth [21].

With improvements in methods and data, the literature has moved forward to address issues, such as confounding, though residual effects still remain given the imperfections in dealing with the statistical challenges and the absence of natural experiments [22]. One possibility that comes close to a natural experiment is the introduction of compulsory health insurance under the UEBMI scheme, which is the focus of this paper.

In this study, we contribute to the literature in several ways. First, we take advantage of the introduction of compulsory health insurance, which eliminates the potential for adverse selection into insurance; we believe this is the first study to assess the effect of China’s UEBMI scheme on infant health outcomes. Second, we use matching methods to match individual characteristics and pregnancy-related health indicators to weaken the effect of potential confounding. Third, we consider the impact of insurance on six distinct infant health outcomes to examine the robustness of the results.

## Methods

### Analytical methods

Four different estimation strategies were employed. We begin with ordinary least squares (OLS) regression and quantile regression (QR) to evaluate the impact of the UEBMI scheme on birth weight (in grams). Then, we estimate probit regression of UEBMI coverage on five other infant health outcomes, which are: premature (< 37 weeks); low birth weight (< 2500 g); very low birth weight (< 1500 g); low Apgar score (< 7 scores); and abnormal health condition at birth (AHCAB).

We then proceed further to use propensity score matching (PSM) to evaluate all six birth outcomes in order to address the potential heterogeneity of insurance. Specifically, we matched women with similar characteristics in order to control for potential confounding, then estimated the independent effect of insurance coverage. We use three matching estimators: one-to-one matching; caliper matching (0.03); and nearest-neighbor matching, to ensure a robust set of estimates. Finally, a probit regression approach was used to analyze the association between insurance coverage under the UEBMI scheme and indicators of maternal health behaviors.

All estimates were performed using STATA 14. For propensity score matching (PSM), we used the “teffects psmatch” command in STATA. While this command allows for the calculation of corrected standard errors of the propensity score estimate, it does not offer balance covariates across treatment groups and within strata. We follow the advice given by Garrido et al. (2014) to use the “psmatch2” command to evaluate the propensity score’s ability to balance covariates across treatment groups and within strata [23].

### Data

This study used medical records data on all women who gave birth between January 1, 2013 and April 30, 2019 at the Shanghai First Maternity and Infant Hospital. The medical data contain patient demographics and clinical details. Maternal health information and abnormal health conditions at birth were coded using the international classification of diseases, 10th revision (ICD-10). This study was approved by the Ethics Committee of Shanghai First Maternity and Infant Hospital.

While there were 160,669 live births over the study period, the analysis sample was restricted by excluding live births where the mother had insurance coverage other than UEBMI coverage, for example, Urban Resident Basic Medical Insurance (URBMI) coverage or private insurance coverage. These restrictions excluded 240 live births and yielded an analysis sample of 160,429 live births. This analysis sample was divided into two mutually exclusive and exhaustive groups: those live births where the mother had UEBMI insurance coverage (*n* = 101,153 live births); and those live births where the mother did not have any insurance coverage, and consequently, covered the entire costs of care themselves (*n* = 59,276 live births). Because migrants that moved to Shanghai to hold occupations of standing may be able to acquire UEBMI coverage, migrants are represented in both groups under review.

### Dependent variable

We estimated the effect of UEBMI coverage on an array of infant birth outcomes and several types of maternal health behaviors as follows.

First, there were six indicators used for infant health outcomes: (1) Birth weight (in grams); (2) Low birth weight, defined as a birth weight below 2500 g (1 = if the baby had a birth weight <  2500 g; otherwise = 0); (3) Very low birth weight, defined as a birth weight below 1500 g (1 = if the baby had a birth weight <  1500 g; otherwise = 0); (4) Low Apgar score, defined as an Apgar score below seven (1 = if the baby had an Apgar score < 7 scores; otherwise = 0); the Apgar score is a test performed shortly after birth to assess the health of a newborn on a scale from 1 to 10; (5) Premature birth, defined as a baby that is born before the completion of 37 weeks of pregnancy (1 = if the baby was born before 37 weeks of pregnancy; otherwise = 0); and an (6) Abnormal health condition at birth (AHCAB), defined as the assignment of any infant diagnostic code at birth by a pediatrician based on use of the International Classification of Diseases, 10th Revision, Clinical Modification (1CD-10-CM), (1 = if the baby had any disease diagnosis, otherwise = 0). An abnormal health condition at birth (AHCAB) includes fetal disease, neonatal malformation, neonatal disease, and other diseases. This includes conditions such as macrosomia, accessory finger(s), undescended testicle, cleft lip, ABO isoimmunization of newborn, congenital pneumonia, neonatal jaundice and other similar infant health conditions. Birth weight is the only continuous variable among the six infant health outcome variables assessed. The other variables were binary.

Second, we used the same set of indicators of maternal health behaviors as those used by Dave et al. (2018) in their assessment of the impact of the Medicaid expansion on maternal health behaviors during pregnancy [10]. These indicators were: gestational diabetes, which may reflect a tendency to be obese; pregnancy-induced hypertension, which may reflect an unhealthy diet; and anemia, which may reflect deficient consumption of iron. We used the same set of indicators for maternal health behaviors to form a binary indicator (1 = the mother has any of the three conditions, gestational diabetes, pregnancy-induced hypertension, or anemia; otherwise = 0) as our dependent variable to estimate whether UEBMI coverage effects pregnancy behaviors. Moreover, we expanded this set of indicators by including two additional indicators: pregnancy complications; and high-risk pregnancy. A high risk of pregnancy arises when the mother or the baby is more likely to have health problems during pregnancy, including medical risk, patients with significant medical and surgical disorders, such as chronic hypertension, cardiac disease, gastrointestinal disease, cancer, HIV/AIDS, sexually transmitted diseases, etc., and obstetric risk, healthy gravidas with fetuses at increased risk of adverse outcomes, such as multiple gestations, prior intrauterine fetal demise, isoimmunization, etc.. Pregnancy complications^1^ refer to a situation wherein medical complications are diagnosed over the course of the pregnancy or at birth. Hence, there are three indicators used for maternal health behavior: (1) Any condition resulting in gestational diabetes, pregnancy-induced hypertension, or anemia (1 = the mother has gestational diabetes, pregnancy-induced hypertension or anemia; otherwise = 0); (2) High risk of pregnancy (1 = being at a high-risk; otherwise = 0); and (3) Pregnancy complications (1 = having pregnancy complications; otherwise = 0).

### Key independent variables

Maternal insurance status was divided into two groups as those covered by the UEBMI scheme and those without any insurance coverage (1 = covered by the UEBMI, 0 = without any insurance coverage). The analysis sample comprised 160,429 live births over this study period with 63.1% having UEBMI coverage (*n* = 101,153 live births), and 36.9% without any insurance coverage (*n* = 59,276 live births).

### Covariate variables and propensity score control variables

In all analyses, we adjusted and matched for demographic factors, maternal clinical characteristics, and pregnancy-related health indicators as follows. We controlled for demographic and relevant health covariates in order to minimize potential selection bias associated with them, including maternal age at the child’s birth, age squared, gravidity - the number of times a woman has been pregnant, birth parity - the number of pregnancies > 20 weeks, ethnicity (1 = Han Chinese; 0 = Ethnic Minority), nationality (1 = China; 0 = others), marriage status (1 = married; 0 = others), employed (1 = employed; 0 = not employed), and mode of delivery (1 = cesarean section; 0 = natural delivery). We also included maternal health indicators for whether a woman had any of three conditions (gestational diabetes, pregnancy-induced hypertension, or anemia), high risk of pregnancy and pregnancy complications as defined previously.

Since having a cesarean section are common and fairly strongly associated with worse pregnancy outcomes, we excluded the cesarean section variable from our PSM methods. As a result, the propensity score control variables are maternal age at the child’s birth, age squared, gravidity, birth parity, ethnicity, nationality, marriage status, employed, maternal health indicators, high risk of pregnancy and pregnancy complications.

## Results

### Data description

Table 1 provides descriptive statistics for each of the variables used in the study. Panel A shows the individual characteristics of the mothers. The average age of the sample of mothers was 30.8 years. The majority (63.1%) had UEBMI coverage, with almost all (94%) of the mothers being employed (The women who had part-time jobs, freelance work or who were self-employed were included in the employed group but they would not have been eligible for UEBMI). The 2.4% of individuals with UEBMI coverage but who were not employed were temporarily out-of-work and still covered by the UEBMI scheme. The overwhelming majority (99%) of the women were married, and most (99%) were Han Chinese. Over 41% of the women had a cesarean delivery. In terms of migrant status, about 39% of mothers were migrants.

**Table 1:** Summary statistics on the characteristics of the study population (those with UEBMI vs. those without any insurance)

	All (*N* = 160,429)		Without any insurance (*N* = 59,276)		With UEBMI (*N* = 101,153)	
Variable	Mean	S.D.	Mean	S.D.	Mean	S.D.
*A: Individual characteristics*						
Age	30.770	3.933	30.780	4.323	30.760	3.686
Age square	962.000	249.200	965.900	271.900	959.800	234.900
Migrant status	0.391	0.488	0.487	0.500	0.334	0.472
Gravidity	1.822	1.102	1.972	1.215	1.734	1.020
Parity	1.256	0.460	1.315	0.506	1.222	0.427
Employed	0.943	0.233	0.886	0.317	0.976	0.154
Ethnic status	0.986	0.117	0.985	0.120	0.987	0.115
Nationality	0.998	0.047	0.994	0.076	1.000	0.007
Marriage status	0.998	0.045	0.996	0.061	0.999	0.031
C-section	0.416	0.493	0.451	0.498	0.395	0.489
*B: Birth outcomes*						
Birth weight (in grams)	3294.000	486.500	3274.000	517.600	3305.000	466.900
Low birth weight (< 2500 g)	0.050	0.218	0.059	0.235	0.045	0.206
Very low birth weight (< 1500 g)	0.00564	0.0749	0.00679	0.0821	0.00448	0.0668
Low Apgar score (< 7 scores)	0.008	0.090	0.010	0.098	0.007	0.085
Prematurity (< 37 weeks)	0.0624	0.242	0.0739	0.262	0.0557	0.229
Abnormal health condition at birth (AHCAB)	0.216	0.412	0.249	0.432	0.197	0.398
*C: Maternal health behavior indicators*						
High-risk pregnancy	0.568	0.495	0.602	0.489	0.548	0.498
Pregnancy complications	0.145	0.352	0.163	0.369	0.135	0.341
Any condition (gestational diabetes, hypertension, anemia)	0.106	0.308	0.131	0.337	0.0914	0.288

The medical records data contain an array of birth outcome measures. Panel B in Table 1 presents descriptive statistics for each of the six birth outcomes. Mean birth weight in the sample was 3294 g. Five percent of the infants had low birth weight (birth weight <  2500 g); approximately 0.6% of the infants had very low birth weight (birth weight <  1500 g); 0.8% of them had a low Apgar score (less than 7 scores); and about 6.24% of the children were born premature (gestational age <  37 weeks). In terms of having an abnormal health condition at birth (AHCAB), there are about 21.6% of the children diagnosed as having an abnormal health condition by pediatricians at birth.

The data set also contains information about prenatal maternal health. Panel C of Table 1 shows summary statistics for pregnancy complications, a high-risk pregnancy, and any of the three conditions (gestational diabetes, pregnancy-induced hypertension, or anemia) being diagnosed by obstetricians over the course of the pregnancy or at birth. About 56.8% of all women experienced a high-risk pregnancy. The magnitude of this percentage may seem high, but a high-risk pregnancy was diagnosed at any time over the course of the pregnancy and includes a wide collection of information on maternal health, particularly past medical and obstetric history, hence, the range of conditions captured and the duration over which such conditions were assessed was large. Almost 14.5% of the mothers were diagnosed with pregnancy complications over the course of their pregnancy or at birth; and approximate 10.6% of the women were diagnosed with any of the three conditions: gestational diabetes, pregnancy-induced hypertension or anemia.

### Determinants of birth weight

We begin by presenting the effects of UEBMI coverage on infant birth weight (in grams) by using ordinary least squares (OLS) and quantile regressions (QR), as shown in Table 2. The OLS regression demonstrates that UEBMI coverage was associated with a significant increase in birth weight by 31.750 g (column 1, *p* <  0.001). The quantile regression (QR) estimates indicate that UEBMI coverage was associated with a significant increase in birth weight at all quantiles: 43.290 g (column 3, *p* <  0.001) at the 10% quantile; 30 g (column 5, *p* <  0.001) at the 25% quantile; 25.096 g (column 7, *p* <  0.001) at the 50% quantile; 23.429 g (column 9, *p* <  0.001) at the 75% quantile; and 15.109 g (column 11, *p* <  0.001) at the 90% quantile. These results demonstrate that UEBMI coverage was associated with an improvement in infant birth weight, but this gain in birth weight was smaller at higher quantiles. Consequently, UEBMI coverage had the most significant gain in birth weight for infants with lower weights.

**Table 2:** OLS and QR the UEBMI coverage effects of infant birth weight

	OLS		0.10QR		0.25QR		0.50QR		0.75QR		0.90QR	
	β	*P*-Value	β	*P*-Value	β	*P*-Value	β	*P*-Value	β	*P*-Value	β	*P*-Value
Variable	(1)	(2)	(3)	(4)	(5)	(6)	(7)	(8)	(9)	(10)	(11)	(12)
UEBMI coverage	31.750^***^	(<0.001)	43.290^***^	(< 0.001)	30.000^***^	(<0.001)	25.096^***^	(<0.001)	23.429^***^	(<0.001)	15.109^***^	(<0.001)
Age	5.114	(0.135)	14.919^*^	(0.026)	6.771	(0.116)	0.178	(0.961)	0.357	(0.926)	0.380	(0.939)
Age square	−0.149^**^	(0.006)	−0.301^**^	(0.004)	−0.164^*^	(0.016)	−0.057	(0.324)	−0.071	(0.243)	−0.082	(0.302)
Employed	13.106^*^	(0.014)	28.987^**^	(0.006)	22.722^***^	(0.001)	4.873	(0.396)	0.143	(0.981)	−6.168	(0.430)
Ethnic status	14.537	(0.182)	23.378	(0.273)	−2.698	(0.844)	1.694	(0.885)	11.714	(0.342)	25.217	(0.113)
Nationality	11.911	(0.663)	95.998	(0.072)	41.684	(0.225)	15.092	(0.607)	9.429	(0.760)	−0.353	(0.993)
Marriage status	99.826^***^	(<0.001)	202.361^***^	(<0.001)	105.324^**^	(0.002)	42.739	(0.144)	58.571	(0.057)	7.120	(0.858)
Migrant status	−11.868^***^	(<0.001)	−26.246^***^	(<0.001)	−9.459^**^	(0.003)	1.087	(0.694)	6.429^*^	(0.027)	6.168	(0.101)
Gravidity	7.714^***^	(<0.001)	1.622	(0.552)	8.562^***^	(<0.001)	11.585^***^	(<0.001)	13.714^***^	(<0.001)	15.652^***^	(<0.001)
Parity	65.915^***^	(<0.001)	112.263^***^	(<0.001)	83.631^***^	(<0.001)	51.430^***^	(<0.001)	41.286^***^	(<0.001)	30.380^***^	(<0.001)
C-section	−41.712^***^	(<0.001)	− 198.378^***^	(<0.001)	−75.541^***^	(<0.001)	−13.016^***^	(<0.001)	35.000^***^	(<0.001)	76.033^***^	(<0.001)
High-risk pregnancy	−29.376^***^	(<0.001)	−114.256^***^	(<0.001)	−53.659^***^	(<0.001)	−20.000^***^	(<0.001)	11.571^***^	(<0.001)	59.864^***^	(<0.001)
Pregnancy complications	101.154^***^	(<0.001)	− 181.431^***^	(<0.001)	−74.947^***^	(<0.001)	68.852^***^	(<0.001)	405.429^***^	(<0.001)	341.277^***^	(<0.001)
Any condition (gestational diabetes, hypertension, anemia)	−49.432^***^	(<0.001)	233.334^***^	(<0.001)	106.834^***^	(<0.001)	−40.654^***^	(<0.001)	− 355.571^***^	(<0.001)	− 293.832^***^	(<0.001)
Constant	3054.148^***^	(<0.001)	2197.607^***^	(<0.001)	2738.961^***^	(<0.001)	3212.806^***^	(<0.001)	3447.714^***^	(<0.001)	3727.391^***^	(<0.001)
Observations (*N*)	160,429		160,429		160,429		160,429		160,429		160,429	

^*^p < 0.05, ^**^ *p* < 0.01, ^***^ *p* < 0.001

### Determinants of various infant health outcomes

We extended the results to other birth outcomes using probit regression methods. The results showing the impact of UEBMI coverage on the incidence of premature birth, low birth weight, very low birth weight, low Apgar score, and having an abnormal health condition at birth (AHCAB) are reported in Table 3. The marginal effects, as shown in Table 3, consistently demonstrate that UEBMI coverage was associated with a significant reduction in: the likelihood of a premature birth, 0.011 (column 1, *p* <  0.001); low birth weight, 0.009 (column 3, *p* <  0.001); very low birth weight, 0.002 (column 5, *p* <  0.001); low Apgar score, 0.002 (column 7, *p* <  0.001); and a reduction in the likelihood of having an abnormal health condition at birth (AHCAB), 0.056 (column 9, *p* <  0.001). Notably, some of the control variables have a statistically significant association with infant birth weight. For instance, parity had negative effects on adverse birth outcomes, which suggests that having more children improved such outcomes. In contrast, detrimental effects on infant health outcomes were found for infants born via cesarean delivery and those where the mother had a high-risk pregnancy.

**Table 3:** Probit regression the UEBMI coverage effects of other five infant health outcomes

	Premature (< 37 weeks)		Low Birth Weight (< 2500 g)		Very Low Birth Weight (< 1500)		Low Apgar Score (<7 scores)		Abnormal health condition at birth (AHCAB)	
	Marginal effects	*P*-value	Marginal effects	*P*-value	Marginal effects	*P*-value	Marginal effects	*P*-value	Marginal effects	*P*-value
Variable	(1)	(2)	(3)	(4)	(5)	(6)	(7)	(8)	(9)	(10)
UEBMI coverage	−0.011***	(<0.001)	− 0.009***	(< 0.001)	− 0.002***	(<0.001)	− 0.002***	(<0.001)	− 0.056***	(< 0.001)
Age	− 0.005**	(0.001)	− 0.003**	(0.005)	− 0.000	(0.508)	− 0.001	(0.052)	0.014***	(< 0.001)
Age square	0.000***	(<0.001)	0.000**	(0.001)	0.000	(0.415)	0.000*	(0.042)	−0.000***	(< 0.001)
Employed	−0.009***	(<0.001)	−0.006**	(0.003)	−0.000	(0.767)	−0.001	(0.308)	0.002	(0.658)
Ethnic status	−0.002	(0.676)	−0.005	(0.241)	−0.001	(0.533)	0.000	(0.830)	0.014	(0.112)
Nationality	0.005	(0.675)	−0.014	(0.241)	0.002	(0.312)	−0.006	(0.320)	−0.054*	(0.031)
Marriage status	−0.028	(0.056)	−0.038**	(0.009)	−0.007	(0.209)	−0.009	(0.173)	−0.024	(0.317)
Migrant status	0.007***	(<0.001)	0.008***	(< 0.001)	0.002***	(<0.001)	0.001*	(0.016)	−0.002	(0.362)
Gravidity	0.004***	(<0.001)	0.001	(0.079)	0.001***	(<0.001)	0.000	(0.250)	0.006***	(< 0.001)
Parity	−0.012***	(<0.001)	− 0.018***	(< 0.001)	− 0.002***	(<0.001)	− 0.002***	(< 0.001)	− 0.064***	(< 0.001)
C-section	0.045***	(<0.001)	0.043***	(< 0.001)	0.003***	(<0.001)	0.001*	(0.011)	0.016***	(< 0.001)
High-risk pregnancy	0.038***	(<0.001)	0.029***	(< 0.001)	0.004***	(<0.001)	0.004***	(< 0.001)	0.073***	(< 0.001)
Pregnancy complications	0.030***	(< 0.001)	0.036***	(< 0.001)	0.003***	(<0.001)	−0.001	(0.196)	0.229***	(< 0.001)
Any condition (gestational diabetes, hypertension, anemia)	−0.036***	(< 0.001)	−0.031***	(< 0.001)	− 0.004***	(<0.001)	−0.004***	(<0.001)	− 0.148***	(< 0.001)
Observations (N)	160,429		160,429		160,429		160,429		160,429	

^*^p < 0.05, ^**^ *p* < 0.01, ^***^ *p* < 0.001

### Propensity score matching (PSM) for all birth outcomes

To reduce the potential for confounding, we used propensity score matching (PSM) methods to compare the women with UEBMI coverage to those without any insurance coverage (and consequently, covered the entire costs of care themselves). We derived separate estimates for each of our six birth outcomes, using three different propensity score matching (PSM) approaches, namely one-to-one matching; caliper matching (0.03); and nearest-neighbor matching, and we summarized the sample sizes for each matching approach as shown in the supplementary material (Table 7 of the Appendix). After matching, the sample size associated with the one on one matching method is 160,411; for caliper matching it is 160,051, and it is 160,411 for nearest-neighbor matching. Since caliper matching is associated with the greatest loss in sample size, we reviewed the mean values of the covariates before and after the matching and assessed the corresponding bias reduction as shown in the supplementary material (Table 8 of the Appendix). After matching, the mothers with UEBMI coverage and those without UEBMI coverage were no longer significantly different in most of these covariates.

The results of propensity score matching (PSM) methods as shown in Table 4. The results of propensity score matching (PSM) methods as shown in Table 4. In each case, the results demonstrate a statistically significant improvement in each of the six infant health outcomes in the presence of UEBMI coverage. We first report the results for the Birth weight Panel; the results show that UEBMI coverage was associated with a significant absolute increase in birth weight of about 30 g to 31 g (rows 1 to 3, *p* < 0.001). The results shown in the Premature birth Panel demonstrate that UEBMI coverage was associated with a significant reduction in the likelihood of premature birth by 0.013 (rows 1 to 3, *p* < 0.001). Similarly, the low birthweight Panel shows that UEBMI coverage was associated with a significant reduction in the probability of having an infant of low birth weight by 0.011 (rows 1 to 3, *p* < 0.001); the very low birthweight Panel demonstrates that UEBMI coverage was associated with a lower probability of having a very low birth weight by 0.003 (rows1 to 3 *p* < 0.001); and the Low Apgar Score Panel demonstrates that UEBMI coverage was associated with a lower probability of having a low Apgar score by 0.002 (rows1 to 3 *p* < 0.001). In terms of having an abnormal health condition at birth (AHCAB), UEBMI coverage was associated with a significantly lower probability of this outcome by 0.054 to 0.055 (rows 1 to 3, *p* < 0.001). Almost all of the propensity score matching methods yield findings that are consistent with the ordinary least squares (OLS) results shown in Tables 2 and 3.

**Table 4:** Three propensity score matching (PSM) approaches the UEBMI coverage effects of all six infant health outcomes

	ATE	*P*-value
Outcome	(1)	(2)
*Birthweight (in grams) Panel*		
One to one	29.601***	(<0.001)
Caliper (0.03)	29.601***	(<0.001)
Nearest Neighbour	31.204***	(<0.001)
*Premature (< 37 weeks) Panel*		
One to one	−0.013***	(<0.001)
Caliper (0.03)	−0.013***	(<0.001)
Nearest Neighbour	−0.013***	(<0.001)
*Low Birthweight (<2500 g) Panel*		
One to one	--0.011***	(<0.001)
Caliper (0.03)	--0.011***	(<0.001)
Nearest Neighbour	--0.011***	(<0.001)
*Very Low Birthweight (< 1500 g) Panel*		
One to one	−0.003***	(<0.001)
Caliper (0.03)	−0.003***	(<0.001)
Nearest Neighbour	−0.003***	(<0.001)
*Low Apgar Score (<7 scores) Panel*		
One to one	−0.002***	(<0.001)
Caliper (0.03)	−0.002***	(<0.001)
Nearest Neighbour	−0.002***	(<0.001)
*Abnormal health condition at birth (AHCAB) Panel*		
One to one	−0.054***	(<0.001)
Caliper (0.03)	−0.054***	(<0.001)
Nearest Neighbour	−0.055***	(<0.001)
Observations (N)	160,429	160,429

^*^p < 0.05, ^**^ *p* < 0.01, ^***^ *p* < 0.001

Notes: The table shows the effect of UEBMI coverage on six birth outcomes. Each panel presents the results of the propensity score matching approach on a separate birth outcome. Birthweight Panel determined the infant birth weight (in grams); Premature Panel for gestational weeks < 37; Low Birth Weight Panel for birth weight < 2500 g; Very Low Birth Weight Panel for birth weight < 1500 g; Low Apgar Score Panel Apgar score determined Apgar score <  7; Abnormal health condition at birth Panel based on the International Classification of Diseases, 10th Revision, Clinical Modification (1CD-10-CM), equres one means if the baby had any disease diagnosis

### The effect of UEBMI coverage on birth outcomes after stratifying by migrant status

Table 5 shows the propensity score matching (PSM) results of the effects of UEBMI coverage on infant health outcomes for the children of migrants relative to those of Shanghai-born women. The same matching variables were used in Table 5 as those used to produce Table 4. Having UEBMI coverage was associated with a significant increase in infant birth weight of 12.4 g for Shanghai-born women (as shown in row 1, column 1 of Table 5, *p* < 0.001), but a much larger increase of 61.777 g for the children born to migrants (row 1, column 3, *p* < 0.001). These results are impressive and signal the large differential impact of UEBMI coverage by migrant status. Other infant health outcomes also demonstrate a differential positive effect of UEBMI coverage on migrants compared to that for locals. Having UEBMI coverage was associated with a small but insignificant increase in the probability of having a premature birth by 0.001 for Shanghai-born women (row 2, column 1, *p* = 0.790), but the reduction was significant and larger for migrants at 0.036 (row 2, column 3, *p* < 0.001). Similar results hold for low birth weight, very low birth weight, and low Apgar scores where the improvement for Shanghai-born mothers was not statistically significant, but the improvement for migrants was larger and statistically significant. For migrants, the UEBMI coverage reduced the probability of having low birth weight by 0.030 (row 3, column 3, *p* < 0.001); reduced the probability of having very low birth weight by 0.009 (row 4, column 3, *p* < 0.001); and reduced the probability of having a low Apgar score by 0.006 (row 5, column 3, *p* < 0.001). In terms of the probability of an abnormal health condition at birth (AHCAB), UEBMI coverage was associated with the statistically significant reduction that was larger for migrants by 0.073 (row 6, column 3, *p* < 0.001) than that for locals by 0.044 (row 6, column 1, *p* < 0.001).

**Table 5:** Propensity score matching (PSM) approaches the UEBMI coverage effects of all six infant health outcomes stratified by maternal migrant status

	Shanghai-native		Migrants	
	ATE	*P*-value	ATE	*P*-value
Outcome	(1)	(2)	(3)	(4)
Birth weight (in grams)	12.400***	(<0.001)	61.777***	(<0.001)
Premature (< 37 weeks)	0.001	(0.790)	−0.036***	(<0.001)
Low birth weight (< 2500 g)	0.001	(0.802)	−0.030***	(<0.001)
Very low birth weight (< 1500 g)	0.001	(0.241)	−0.009***	(<0.001)
Low Apgar score (< 7 scores)	0.001	(0.248)	−0.006***	(<0.001)
Abnormal health condition at birth (AHCAB)	−0.044***	(<0.001)	−0.073***	(<0.001)
Observations (N)	97,716		62,713	

^*^p < 0.05, ^**^ *p* < 0.01, ^***^ *p* < 0.001

### The effect of UEBMI on maternal health behavior during pregnancy

Health insurance may improve infant health outcomes through improvements in maternal health behaviors. Consequently, we tested for the effect of UEBMI coverage on each of the three separate maternal health behavior indicators. The results of the marginal effects are shown in Table 6. Column 1 of Table 6 demonstrates that UEBMI coverage was associated with a reduction in the occurrence of a pregnancy complication by 0.03 (as in column 1 of Table 6, *p* < 0.001). In the case of a high-risk pregnancy, it was shown that UEBMI coverage was associated with a reduction in the likelihood of a high-risk pregnancy by 0.032 (column 3, *p* < 0.001). Similarity, the UEBMI coverage was shown to be associated with a reduction in the likelihood of having any of the three conditions (gestational diabetes, pregnancy-induced hypertension, or anemia) by 0.045 (column 5, *p* < 0.001).

**Table 6:** Probit regression the UEBMI coverage effects of three different pregnancy-related health indicators

	Pregnancy complications		High-risk pregnancy		Any condition (gestational diabetes, hypertension, anemia)	
	Marginal effects	P-value	Marginal effects	*P*-value	Marginal effects	P-value
Variable	(1)	(2)	(3)	(4)	(5)	(6)
UEBMI coverage	−0.030***	(<0.001)	− 0.032***	(<0.001)	−0.045***	(<0.001)
Age	0.011***	(<0.001)	−0.009*	(0.023)	0.010***	(<0.001)
Age square	−0.000	(0.200)	0.000***	(<0.001)	−0.000	(0.251)
Employed	0.004	(0.253)	0.011*	(0.049)	0.010**	(0.002)
Ethnic status	0.007	(0.398)	−0.060***	(<0.001)	0.007	(0.326)
Nationality	0.029	(0.083)	0.037	(0.223)	0.018	(0.219)
Marriage status	−0.018	(0.409)	0.012	(0.669)	0.015	(0.367)
Migrant status	−0.019***	(<0.001)	0.064***	(<0.001)	−0.022***	(<0.001)
Gravidity	0.008***	(<0.001)	0.016***	(<0.001)	0.002**	(0.003)
Parity	−0.033***	(<0.001)	−0.007*	(0.046)	−0.027***	(<0.001)
C-section	0.024***	(<0.001)	0.256***	(<0.001)	−0.006***	(<0.001)
Observations (N)	160,429		160,429		160,429	

*p*-values in parentheses

^*^p < 0.05, ^**^ *p* < 0.01, ^***^ *p* < 0.001

### Sensitivity analysis

**Fig. 1:**
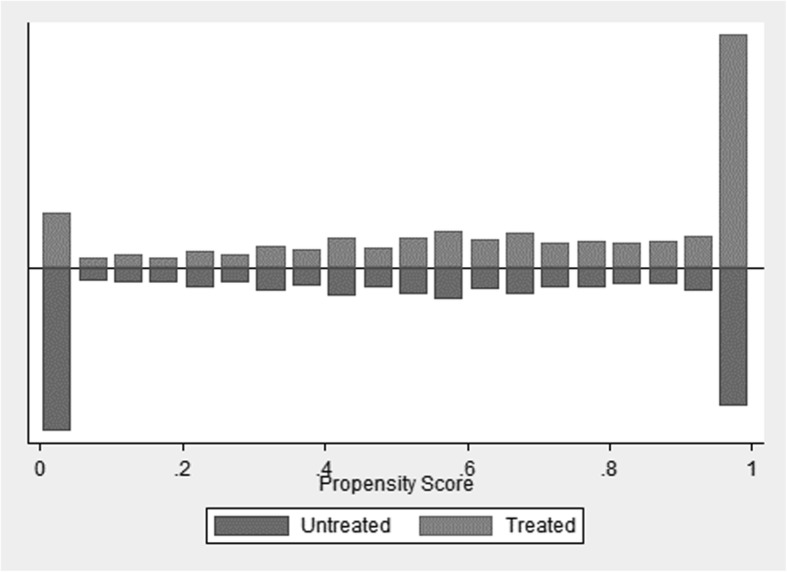
Propensity score histograms by treatment status (untreated = without any insurance, treated = with UEBMI coverage)

We examined how robust our results were to alternative estimation techniques and how the results may have changed with modification to the definition of some variables. First, in the case of each of the three alternative matching methods, all the results were very similar (Table 4) and the results were also close to the results using ordinary least squares (OLS) regression and quantile regression (QR), as shown in Table 2, and probit regression, as shown in Table 3. Second, we also examined whether our results were maintained when we stratified by migration status. The results demonstrated that the subgroup of migrants was statistically significant and yielded findings consistent with the other results reported herein. Third, we graphed the histograms of the propensity score by treatment (insurance) status, as shown in the supplementary material (Figure 1 of the Appendix). It can be seen intuitively from the figure that the distribution of propensity score values between the treated group (with UEBMI coverage) and the untreated group (without any insurance) is well-balanced.

## Discussion

### Key findings and implications

Evaluating whether health insurance coverage would improve infant health is a challenge as the issues of potential adverse selection and heterogeneity into insurance groups need to be overcome. This study addressed this question by estimating the effect of China’s compulsory insurance scheme (UEBMI) on six separate infant birth outcomes while using propensity score matching methods to control for potential confounding. Our analysis yields three key findings.

First, UEBMI coverage was shown to be associated with improvements in all six of the infant birth outcomes assessed. Specifically, the results suggest that UEBMI coverage was associated with: an increase in birth weight of about 30 g; a reduction in the incidence of having a premature birth by 0.013; a reduction in the incidence of having a child with low birth weight by 0.011; a reduction in the incidence of having a child with very low birth weight by 0.003; a reduction in the incidence of having a low Apgar score by 0.002, and a reduction in the incidence of having a baby diagnosed with the disease at birth by 0.054 to 0.055. Our findings also reveal that UEBMI coverage had the most significant gain in birth weight for infants of lower weight. These findings were in keeping with the main theme in the literature. Specifically, the significance and direction of the coefficients on UEBMI coverage on birth weight, low birth weight, very low birth weight, low Apgar score, and having a premature birth were consistent with previous findings [2, 6, 13, 14, 20, 21, 24, 25]. While there is a paucity of literature that has examined the association between health insurance and the incidence of an abnormal health condition at birth (AHCAB), possibly due to an absence of data, our results are consistent with the rest of our findings showing positive infant birth outcomes with insurance coverage.

Second, after stratifying by maternal migrant status, UEBMI coverage was shown to have a greater effect on migrant mothers compared to local residents of Shanghai. Specifically, we found that UEBMI coverage increased the birth weight by 61.777 g for the babies of migrants compared to 12.4 g for the babies of local residents of Shanghai. The direction and magnitude of results for the other five infant birth outcomes were similar. The underlying health condition of migrants was much worse than that for urban residents in China, which is a major policy issue within the academic community and government [9, 26]. Some of the studies suggested that a lack of urban health insurance may as a crucial factor in health inequality between migrants and urban residents [27, 28]. Our findings offer evidence to show that the health of migrants may dramatically improve once they have UEBMI coverage.

Third, we found evidence that UEBMI coverage was associated with improvements in maternal health behaviors. Specifically, our results demonstrated that UEBMI coverage significantly reduced the likelihood of having pregnancy complications by 0.030, was associated with a reduction in the likelihood of having a high-risk pregnancy by 0.032, and was associated with a reduction in the likelihood of having any of the following three conditions (gestational diabetes, pregnancy-induced hypertension, or anemia) by 0.045. Increasing maternal health behavior during pregnancy is one of the possible channels through which health insurance may improve infant birth outcomes. Our findings were consistent with the way in which health insurance may shift maternal health behaviors that result in improvements in infant birth outcomes.

### Limitations

There are a range of limitations to highlight. First, our study was based on inpatient data from a single hospital in Shanghai, which may limit the generalizability of the findings. While single-center studies pose challenges for generalizability, this hospital is situated in one of the most diverse regions of China and the women included in the study were diverse in clinical, demographic and ethnic background, which helps to improve generalizability. Moreover, the study hospital is one of the largest obstetrical hospitals in China and is associated with the largest annual number of births nationwide at more than 30,000 births.

Second, while the information used for this study was drawn from hospital administrative data that comprised comprehensive maternal medical diagnostic and demographic data, there were some variables that were not captured in that database and consequently, there is the possibility of omitted variable and misspecification bias. However, we used six different birth outcomes (birth weight, premature birth, low birth weight, very low birth weight, low Apgar score, and infant abnormal health conditions at birth) and a range of estimation techniques to ensure that our estimates were robust. Consequently, we believe that these concerns, if still present, maybe small.

Third, the study offers estimates of the effects of insurance after controlling for a range of potential confounding variables, yet, unmeasured and residual confounding may still persist. While our results are robust to each of the propensity score approached assessed, we cannot exclude the possibility that some remaining confounding might persist for all of those estimation approaches.

Finally, we were unable to directly test for the impact of a change in specific maternal health behaviors, such as smoking and drinking, during pregnancy when they had UEBMI coverage. This absence of information limited our interpretation of the channels through which UEBMI coverage might improve infant birth outcomes to those associated with “black box” changes associated with the maternal health behaviors explored, e.g., high-risk pregnancies, pregnancy complications, and has any of the following three conditions: gestational diabetes; pregnancy-induced hypertension; or anemia. Further research is needed to garner a more complete picture of women’s behavior during pregnancy.

## Conclusion

This paper demonstrated the UEBMI coverage was associated with improvements in infant birth outcomes after using propensity score matching to deal with potential confounding. This finding was evident in each of six different birth outcomes (birth weight, premature birth, low birth weight, very low birth weight, low Apgar score, and an abnormal health condition at birth). When subgroup analyses were conducted, there were larger potential gains in infant health outcomes among migrant mothers than their local counterparts. We also explored possible mechanisms through which health insurance might impact infant health and demonstrated that UEBMI coverage tended to alter maternal health behaviors, such as reducing the possibility of having a pregnancy complication, a reduction of the likelihood of a high-risk pregnancy, and a reduction of the possibility of having any of the following three conditions: gestational diabetes; pregnancy-induced hypertension; or anemia. Our findings suggest that health insurance coverage for pregnant women, especially for migrant mothers, have the potential to directly and significantly improve infant health outcomes. Further research is required to determine whether the effectiveness findings can be replicated for other Chinese jurisdictions, and a detailed understanding of the cost-effectiveness of a policy to expand insurance coverage is warranted.

## Data Availability

Our data will not be shared to protect the participants’ anonymity.

## Appendix

**Table 7:** Summary for the number of individuals off and on support

	Common support		
Treatment assignment	Off Support	On Support	Total
One on one			
Untreated	16	59,260	59,276
Treated	2	101,151	101,153
Total	18	160,411	160,429
Caliper (0.03)			
Untreated	374	58,902	59,276
Treated	4	101,149	101,153
Total	378	160,051	160,429
Nearest Neighbour			
Untreated	16	59,260	59,276
Treated	2	101,151	101,153
Total	18	160,411	160,429

**Table 8:** Mean values of the covariates before and after the matching (caliper matching)

		Mean				t-test	
Variable	Sample	Treated	Control	%bias	%|bias| reduction	t	*p* > |t|
Age	Unmatched	30.76	30.78	−0.40		− 0.76	0.45
	Matched	30.76	30.74	0.60	−47.50	1.39	0.17
Age square	Unmatched	959.80	965.90	−2.40		−4.70	<0.001
	Matched	959.80	958.30	0.60	74.50	1.48	0.14
Migrant	Unmatched	0.33	0.49	−31.40		−61.24	<0.001
	Matched	0.33	0.34	−0.80	97.60	−1.77	0.08
Gravidity	Unmatched	1.73	1.97	−21.20		−41.98	<0.001
	Matched	1.73	1.73	0.50	97.40	1.37	0.17
Parity	Unmatched	1.22	1.32	−19.90		−39.27	<0.001
	Matched	1.22	1.22	0.30	98.40	0.78	0.43
Employed	Unmatched	0.98	0.89	35.70		75.45	<0.001
	Matched	0.98	0.98	0.01	100.00	−0.06	0.95
Ethnic status	Unmatched	0.99	0.99	1.10		2.05	0.04
	Matched	0.99	0.99	−1.20	−17.20	−2.93	<0.001
Nationality	Unmatched	1.00	0.99	10.70		23.92	<0.001
	Matched	1.00	1.00	0.01	99.70	0.58	0.56
Marriage status	Unmatched	1.00	1.00	5.90		12.33	<0.001
	Matched	1.00	1.00	−0.40	92.70	−1.62	0.11
High-risk pregnancy	Unmatched	0.55	0.60	−11.00		−21.30	<0.001
	Matched	0.55	0.55	−0.40	96.70	−0.80	0.42
Pregnancy complications	Unmatched	0.14	0.16	−7.90		−15.52	<0.001
	Matched	0.14	0.13	0.40	94.90	0.95	0.34
Any condition (gestational diabetes, hypertension, anemia)	Unmatched	0.09	0.13	−12.60		−24.80	<0.001
	Matched	0.09	0.09	0.80	93.70	1.94	0.05

## Abbreviations

AHCAB: Abnormal health condition at birth
BW: Birth weight
DID: Difference in differences approach
FE: Fixed effects
GA: Gestational age
ICD-10-CM: International Classification of Diseases, 10th Revision, Clinical Modification
IV: Instrumental variables
LBW: Low birth weight
NCMS: New Cooperative Medical Scheme
OLS: Ordinary least squares regression
PSM: Propensity score matching
QR: Quantile regression
UEBMI: Urban Employee Basic Medical Insurance
URBMI: Urban Resident Basic Medical Insurance coverage
VLBW: Very low birth weight
WIC: Special Supplemental Nutrition Program for Women, Infants, and Children

## Footnotes

1 Pregnancy complications ICD-10-CM codes O44.0 O44.1 O13.1 O13.2 O13.3 O13.9 O14.0 O48.0 O26.2 O99.3 O24.4 O26.8 O30.0 O36.0 O36.5 O36.6 O24.9; gestational diabetes mellitus ICD-10-CM codes O24.9.
